# The Effects of Mindfulness-Focused Internet-Based Cognitive Behavioral Therapy on Elevated Levels of Stress and Symptoms of Exhaustion Disorder: A Randomized Controlled Trial

**DOI:** 10.32872/cpe.12899

**Published:** 2024-09-30

**Authors:** Kristofer Vernmark, Timo Hursti, Victoria Blom, Robert Persson Asplund, Elise Nathanson, Linda Engelro, Ella Radvogin, Gerhard Andersson

**Affiliations:** 1 Department of Behavioural Sciences and Learning, Linköping University, Linköping, Sweden; 2 Department of Psychology, Uppsala University, Uppsala, Sweden; 3 The Swedish School of Sport and Health Sciences, Stockholm, Sweden; 4 Psykologpartners AB, Linköping, Sweden; 5 Department of Biomedical and Clinical Sciences, Linköping University, Linköping, Sweden; 6 Department of Clinical Neuroscience, Karolinska Institute, Stockholm, Sweden; Philipps-University of Marburg, Marburg, Germany

**Keywords:** CBT, ICBT, stress, mindfulness, exhaustion, internet-based

## Abstract

**Background:**

Internet-based Cognitive Behavior Therapy (ICBT) and mindfulness interventions are commonly used to treat elevated levels of stress. There are however few high-quality studies that examine ICBT with integrated mindfulness components for symptoms of stress and exhaustion, and the role of mindfulness exercises in digital treatment.

**Method:**

The aim of the present study was to evaluate if a mindfulness-focused ICBT-program could reduce symptoms of stress and exhaustion, and increase quality of life, in a randomized controlled trial including 97 self-referred participants between 18 and 65 years who experienced elevated levels of stress.

**Results:**

The intervention group had significantly reduced symptoms of stress and exhaustion, and increased quality of life, compared to the control group. Compared with the controls, participants in the intervention group showed a significant improvement with moderate to large effects on the primary outcome measure perceived stress (*d* = 0.79), and the secondary outcomes, exhaustion (*d* = 0.65), and quality of life (*d* = 0.40). Participants in the ICBT group also increased their level of mindfulness (*d* = 0.66) during the program. The amount of mindfulness training was significantly associated with an increased level of mindfulness, which in turn was significantly associated with reduced stress symptoms.

**Conclusions:**

Mindfulness-focused ICBT can be an effective method to reduce stress-related mental health problems and the amount of mindfulness training seems to be of importance to increase the level of experienced mindfulness after treatment.

## Highlights

- Combining mindfulness with ICBT is effective when treating individuals with elevated stress.
- Mindfulness-based ICBT can decrease symptoms of exhaustion and increase quality of life.
- The amount of mindfulness training during treatment has an impact on self-rated mindfulness.
- Increased mindfulness is associated with decreased levels of stress.

Perceived stress is conceptualized as an individual’s feelings or thoughts about how much stress one is experiencing at a given point in time or during a period of time. To experience stress is a fundamental feature of human beings and can be an adaptive response to various stressors in everyday life. Stress becomes an issue when the body is forced to mobilize energy for an extended period of time without sufficient recovery. During the last decades, rapid advancements in society have transformed how we live, work, and interact, resulting in higher exposure to mental strain (Atroszko et al., 2020). Elevated stress over longer periods of time can have a negative impact on daily life and is associated with other physical and psychological problems such as anxiety, impaired sleep, depression, and exhaustion (Cohen et al., 2007; Grossi et al., 2015). Increased levels of stress are also associated with a negative impact on quality of life (Parsaei et al., 2020), a wider construct of self-perceived satisfaction with important life areas such as work, friendship, creativity, and leisure, that should be measured separately from other mental health symptoms (Lindner et al., 2016). Consequently, stress-related health issues have increasingly been recognized as a significant health issue with prevalence ranging between 4% and 16% (Glise et al., 2010; Höglund et al., 2020). Even though the most common diagnostic manuals ICD-11 and DSM-V contain definitions and categorizations such as post-traumatic stress, acute stress reaction and adjustment disorder, there is still a lack of consensus and well-defined terminology related to stress induced problems that are associated with relational conflicts, economic hardship, or work-related stressors. Another diagnosis close to stress-related disorders is Exhaustion Disorder (ED), which is similar to the concept of clinical burnout (van Dam, 2021). It is characterized by severe mental and physical fatigue, in combination with lack of initiative and endurance. Mental and physical effort in daily activities lead to long recovery periods and it is a common cause for workplace sick leave in Sweden (Lindsäter et al., 2022). Prolonged exposure to stress has direct effects on people’s well-being and leads to immense costs for society (Grossi et al., 2015; Hassard et al., 2018; Kivimäki & Steptoe, 2018; Melchior et al., 2007). Despite these well-known and detrimental consequences, a majority of all individuals suffering from stress and other mental health-related disorders, remain untreated (Ebert et al., 2016). This calls for further development and evaluation of interventions that are accessible, cost-effective, and have the potential in reducing stress.

Two established methods for the treatment of mental health problems are Cognitive Behavior Therapy (CBT) and Mindfulness interventions. CBT is considered an evidence-based and cost-effective treatment method for common mental health problems that is often provided individually in face-to-face settings (Bhattacharya et al., 2023; Butler et al., 2006; Myhr & Payne, 2006). It incorporates behavioral and cognitive strategies with the addition of homework assignments between sessions (Wenzel et al., 2016). To this date it is the most researched psychotherapy method, although its effects for stress related problems have been less studied (Nakao et al., 2021). Mindfulness has its roots in Buddhist traditions and can be described as the psychological process of purposefully focusing attention on experiences occurring in the present moment (Kabat‐Zinn, 2003). The ability to be mindful is considered a skill that can be trained through practice (Bishop et al., 2004) and mindfulness-based interventions are commonly used to increase wellbeing and treat mental health problems (Sverre et al., 2023). There are also correlational studies examining the role of mindfulness in relation to stress and quality of life (Javaid et al., 2023), some of which are showing that higher levels of present moment awareness and mindful attention can lead to lower levels of perceived stress and increased wellbeing (Hepburn et al., 2021). Further examinations of the amount and duration of mindfulness training and its effects on dispositional mindfulness scale measurements are also warranted (Quaglia et al., 2016).

There is a growing body of evidence to support the efficacy of stress management interventions (SMI) in different populations and on a wide range of outcomes, such as perceived stress, burnout, recovery, and quality of life (Bhui et al., 2012). SMIs based on CBT, have yielded the largest effect sizes (Cohen's *d* = 1.16), followed by mindfulness and relaxation-based interventions (Cohen's *d* = 0.50; Richardson & Rothstein, 2008). Common components in stress-focused CBT vary but interventions commonly include a rationale about stress and how to manage stressors, relaxation techniques, coping and activation techniques, cognitive restructuring, problems solving, and skills training in assertiveness and time management (Ghazavi et al., 2016).

Mindfulness-based stress reduction, focusing on mindfulness techniques (e.g., directed attention to bodily sensations, thoughts, feelings, and daily activities) has been found to reduce stress symptoms in non-clinical and clinical samples (Chiesa & Serretti, 2009; Shapiro et al., 2005; Smith et al., 2008) as well as in employees (Janssen et al., 2018) and students (Deshpande et al., 2023). Mindfulness has shown similar effects as CBT and pharmacological treatment for stress symptoms (Khoury et al., 2013). Mindfulness has also been incorporated into modern third-wave cognitive behavior therapy approaches, such as Acceptance and Commitment Therapy (ACT; Hayes, 2016), where it is used together with other concepts such as values and acceptance, aimed at increasing psychological flexibility and quality of life. There is data showing that incorporating value-based action in mindfulness interventions could enhance the effects of mindfulness (Christie et al., 2017). Previous trials (e.g., Michel et al., 2014) have also suggested that mindfulness could be an effective segmentation strategy to promote work–life balance for employees struggling with stress-related rumination and psychological preoccupation with work concerns. Although studied separately, few studies have examined the combined effects of integrated CBT and mindfulness-based stress reduction interventions.

Internet-based CBT (ICBT) is a well-established treatment format that offers increased access to effective psychological interventions for a wide range of mental health problems (Andersson, Titov, et al., 2019). Since one of the first studies on internet-based stress interventions (Zetterqvist et al., 2003) a growing body of literature has provided evidence of the efficacy in various populations (Andersson, Carlbring, et al., 2019). It has several advantages compared to face-to-face CBT, including being cost-effective by consuming less therapist time and reducing waiting times (Catarino et al., 2023), as well as being less emotionally stressful and bringing variety to a therapist’s daily work (Weineland et al., 2020). The effects are long-lasting (Andersson et al., 2018) and similar to face-to-face treatment (Hedman‐Lagerlöf et al., 2023). Meta-analyses have yielded small to moderate effects on outcomes of perceived stress, burnout, exhaustion, depression, and anxiety (Heber et al., 2016; Svärdman et al., 2022). Subgroup analyses have revealed greater improvement in guided interventions (Heber et al., 2017) and recent trials have suggested that ICBT stress interventions could have long-lasting effects (12 months post-treatment) and accelerate recovery and return to work (Asplund et al., 2023).

There are examples of studies on other disorders that have added mindfulness components in ICBT-programs (Carlbring et al., 2013) but to our knowledge few studies have evaluated the full integration of CBT and mindfulness components delivered in a concise internet-based format for perceived stress and symptoms of exhaustion. There is also a need for further knowledge about the association between the amount and length of mindfulness training in shorter treatment programs, and if increased mindfulness is associated with lower levels of perceived stress. The aim of the present study was to evaluate a six-week mindfulness-focused ICBT program for stress and its effects on stress, exhaustion, quality of life, and mindfulness, and the impact of mindfulness training in reducing stress and increasing experienced mindfulness. We hypothesized that the internet-based recovery program would produce greater improvements in perceived stress (primary outcome) compared with a waitlist control group. We also hypothesized that the intervention group would differ with regard to stress-related exhaustion and quality of life. Finally, we hypothesized that the ICBT mindfulness training would be associated with increased mindfulness levels and reduction in perceived stress.

## Method

### Design

In this randomized controlled trial, participants were randomized to an internet-based mindfulness-focused ICBT program or a waitlist control group (WLC). The study followed Consolidated Standards of Reporting Trials (CONSORT) guidelines (Schulz et al., 2010) and was conducted between January 2017 and March 2017. Estimates of sample size were based on calculations in previous controlled trials on ICBT for stress (Ly et al., 2014) where a minimum of 66 participants was needed to achieve a power of 0.80 and detect an effect size of *d* = 0.50 (α level = .05). Self-report outcome measures were collected at pre- and post-treatment (six weeks). Participants who met the study criteria and provided informed consent were allocated randomly by an independent researcher using an online random generator (www.randomizer.org). Participants were randomized to either intervention or to a waitlist control condition. In addition to the pre- and post-assessment, participants reported the intensity of their mindfulness training every week. The study was part of a larger project investigating ICBT for stress and ethical approval was obtained from the local ethics committee (Reference No. 353-31).

### Participants and Recruitment

**Figure 1:**
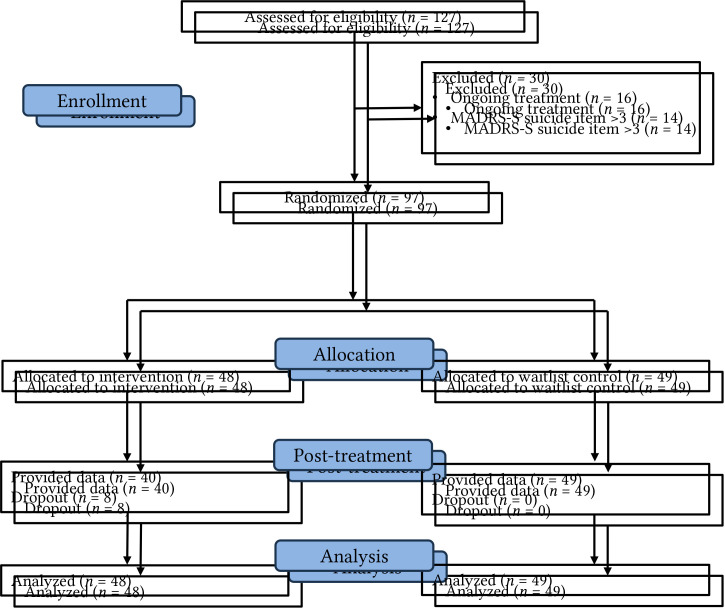
Flow Diagram of Participants in the Study

Participants were recruited by self-referral. Information was distributed by information on websites and social media and by emailing student health centers and human resource staff in some organizations. Those interested in participating were invited to contact the research team by email to receive further information about the study and a link leading to a website with information about the study. Following the link enabled the possibility to give informed consent and answer questions for screening purposes as well as the forms included in the pre-treatment assessment. The questionnaire also included questions about if participants had been diagnosed with exhaustion disorder or another psychiatric diagnose in routine care. Exclusion criteria were ongoing alcohol or drug abuse, ongoing psychological treatment, and a rating of 3 or higher on item 9 (life desire) on the Montgomery Åsberg Depression Rating Scale (MADRS-S; Svanborg & Åsberg, 2001), indicating suicidal ideation. Of the 127 persons completing this phase, 30 were excluded mainly due to ongoing treatment or suicidal ideation. The flowchart of the study is displayed in Figure 1. Individuals with suicidal ideation that were excluded from the study were informed about appropriate help within the Swedish health care system. Participants did not receive any compensation for their participation in the study.

In total 97 persons from different parts of Sweden were included. Inclusion criteria were an age between 18 and 65 years, fluency in Swedish, basic computer skills, and the subjective experience of elevated levels of stress. The sample consisted predominantly of females with a mean age around forty years. Roughly one out of four participants reported that they had been diagnosed with stress-induced exhaustion disorder and among other self-reported diagnoses, depression was most prevalent. More detailed demographic information about the sample is shown in Table 1. During the intervention phase eight participants (8.2%) in the treatment group dropped out and none in the control group.

**Table 1:** Demographic Characteristics of Participants at Pre-Treatment

Baseline characteristics	Intervention group (*n* = 48)		Control group (*n* = 49)	
	*M*	*SD*	*M*	*SD*
**Age**	38.33	11.13	42.22	10.78
	*n*	%	*n*	%
Sex				
Female	41	85	42	86
Male	7	15	7	14
Comorbidity^a^				
Exhaustion disorder	12	25	14	29
Depression	8	17	7	14
Depression and GAD	5	10	6	12
Sleep disorder	1	2	1	2
Bipolarity and Panic disorder	1	2	0	0
Occupation				
Student	9	19	3	6
Full-time employee	23	48	32	65
Part-time employee	6	13	6	12
Job seeker	1	2	1	2
Student and employee	3	6	4	8
Sick leave (full time)	4	8	2	4
Sick leave (part time)	2	4	2	4
Level of education				
Elementary school	0	0	1	2
Upper secondary education, 1-2 years	0	0	4	8
Upper secondary education, 3-4 years	14	30	10	20
University- or college education, 3 years or less	9	18	8	16
University- or college education, >3 years or more	25	52	26	53
Country region				
Northern Sweden	8	17	16	33
Central Sweden	24	50	20	40
Southern Sweden	13	27	11	22
Overseas	3	6	2	4

^a^Self-rating in pre-treatment questionnaire of being diagnosed in routine care.

### Primary Outcome Measure

The Perceived Stress Scale (PSS; Cohen et al., 1983) was used as primary outcome measure to assess the level of experienced stress. The PSS measures the degree to which situations in one’s life are being perceived as stressful. The version used in the study had 14 items which focus on perceived stress during the last month. Individual scores can range from 0 to 56 with higher scores indicating higher perceived stress. The Swedish version of PSS-14 has shown to have good internal consistency (α = .84 – .90; Eklund et al., 2014). Versions of the scale are commonly used in research studies and it has been shown to be responsive to psychological treatment in internet-based interventions for stress (Svärdman et al., 2022; Zetterqvist et al., 2003). While normative population data for Sweden is unavailable, studies conducted in other countries have indicated mean scores ranging between 20.93 and 25.63 for the PSS-14 in various non-clinical sample groups (González-Ramírez et al., 2013).

### Secondary Outcome Measures

Karolinska Exhaustion Disorder Scale (KEDS; Besèr et al., 2014) was used to assess exhaustion related consequences of prolonged stress. KEDS is designed to measure symptoms typical in exhaustion disorder, such as exhaustion, cognitive problems, poor sleep and reduced tolerance to further stress. The scale has 9 items, and each item is answered on a 7-point scale (0 – 6). Verbal descriptions are given to answering alternatives 0, 2, 4, and 6. The maximum score is 54 while the cut-off score for incidence of exhaustion disorder is 19. KEDS has good overall psychometric properties including excellent internal consistency (Cronbach’s alpha, α = .94). Further, KEDS is able to discriminate between exhaustion, depression and anxiety (Besèr et al., 2014).

Brunnsviken Brief Quality of Life (BBQ; Lindner et al., 2016) is a self-rating scale for assessment of subjective quality of life. It consists of 12 statements within six areas of life considered relevant for the experience of quality of life. All items are answered on a 5-point scale (0 = don’t agree at all to 4 = agree completely), but for a given area of life, satisfaction is rated first and then the importance of the area. Afterwards, scores for satisfaction and importance within respective area are multiplied. The maximum score is 96 implying a high level. The psychometric evaluation of the scale suggests good concurrent and convergent validity. Internal consistency is adequate (Cronbach’s alpha, α = .76) and test-retest reliability is high (ICC = .82).

The Five Facets Mindfulness Questionnaire – Swedish version (FFMQ-SWE; Baer et al., 2008; Lilja et al., 2011) has been developed to assess the level of mindfulness. FFMQ-SWE has 29 items distributed on five subscales: Nonreactivity to inner experience, Observing, Acting with awareness, Describing and Nonjudging of experience. The items are rated on a 5-grade likert scale (1 = never or very seldom to 5 = always). The internal consistency for the global scale is good (Cronbach’s alpha, α = .81). and it also has a high content validity (Lilja et al., 2011).

Montgomery Åsberg Depression Rating Scale (MADRS-S; Svanborg & Åsberg, 1994) is a widely used self-rating scale measuring depression. In this study, only item nine was used to allow exclusion due to suicidal ideation or low level of life desire.

In addition to the evaluation of treatment effects, the participants’ experience considering the functionality of the internet treatment system was assessed by The System Usability Scale (SUS; Brooke, 1996). SUS is a 10-item scale composed of 10 statements scored on a 5-point scale with a final score ranging from 0 to 100. A higher score on the scale indicates better usability of a given product or service. A large empirical evaluation of SUS (Bangor et al., 2008) provided support for the validity of the scale as well as guidelines for the interpretation of the results. A Swedish version of the scale have existed and been used since 2011. There are currently no publications on the Swedish version, but translations to other languages have shown retained psychometric properties and conceptual equivalence (Hvidt et al., 2020).

### The Intervention

The treatment program, *Stresshjälpen,* was developed by the private Swedish psychology company Psykologpartners. It contains CBT-components for the treatment of stress-related problems such as psychoeducation about stress, functional analysis of stressful situations, strategies for self-care and healthy habits, time-management strategies, how to handle perfectionism and setting up a plan for setbacks. Mindfulness components and weekly mindfulness exercises are integrated into the treatment from module two and onwards. The modules and their content are further described in Table 2. The intervention is delivered through six online modules containing text, video, audio, and free-form text input boxes. In the beginning of each new module, participants were asked to reflect upon the previous module and how they managed the homework assignments. To keep track of the participants’ mood and motivational level, a few short questions in the end of each module were included covering quality of life, stress, compliance with the treatment and sleep problems.

**Table 2:** Description of the Content in Stresshjälpen

Module	Content
Module 1 – What is stress?	Psychoeducation about stress Stressful situations Reactions in stressful situations Homework: map current experiences of stress and coping strategies, and a 7-day diary with ratings of stress level
Module 2 – Functional analysis and mindfulness	Functional analysis of stressful situations Introduction to mindfulness Homework: functional analysis and mindfulness exercises
Module 3 – Mindfulness and values	Mindfulness Values and valued living Homework: values in specified life domains and mindfulness exercises
Module 4 – Self-care	Committed action Healthy habits: sleep, exercise, and healthy eating Mindfulness to create healthy habits Homework: change an important habit
Module 5 – Time-management and setting boundaries	Time-management strategies Values, functional analysis and strategies for setting boundaries Homework: setting boundaries and mindfulness exercises
Module 6 – Maintaining treatment effects	How to handle perfectionism Summarize the treatment: lessons learned, obstacles and important behaviors Create a maintenance plan and a plan for setbacks Valued living

The two therapists had access to the treatment content in advance and could familiarize themselves with the material. Therapists also underwent a two-day training program in internet-based CBT and had regular supervision with an ICBT-proficient clinical psychologist during the whole study. The study participants were instructed to complete one module per week, and they had access to the treatment website for eight weeks. Two-factor authentication was used for logging in and accessing the material. The communication between the therapists and the participants took place via a secure messaging function in the portal. Once a week the participants received a message with comments on their previous work and further instructions. Therapists aimed to motivate participants, validate and reinforce functional behavior, answer questions and solve problems. Participants who had not been logged in for a longer period were reminded by email or telephone.

### Procedure

There was no face-to-face contact between therapists and participants and all activities were conducted online. The intervention was delivered through a website and the assessments were done via an encrypted website. The randomization via randomizer.org resulted in 48 participants in the intervention group and 49 in the waitlist control group. The treatment started in January 2017 and continued to March 2017. After the post-assessment, participants in the control group were offered the online treatment.

All data analyses were carried out using IBM SPSS Statistics v.29 software. Pretreatment differences on demographic and outcome variables were analyzed with *t*-test and chi^2^-test. Intention to treat (ITT) was employed by using multiple imputation to handle missing data including 20 imputations, as recommended by Enders (Enders, 2017). Analysis of Covariance (ANCOVA) was used with pre-treatment scores as covariates for all the self-report measures to investigate treatment effects (Vickers & Altman, 2001). Effect sizes with Cohen’s *d* with confidence intervals were calculated based on the post-treatment imputed means. The relationship between mindfulness training (assessed weekly) and the change in the level of mindfulness from pre- to post-treatment was analyzed using Spearman’s Rho, due to the skewed distribution of the number of training sessions and the total duration of training. The association between changes in mindfulness levels and experienced stress was analyzed using the Pearson correlation coefficient.

## Results

### Baseline Differences, Ratings of Usability, and Adherence

There were no differences between the study groups at the pretreatment considering demographic or outcome variables. The overall rating for the internet treatment system assessed by SUS was 86 points which is considered excellent (Bangor et al., 2008).

The average number of completed modules in the intervention group was 4.7 of six modules (*SD* = 2.26, range 0-6) and 71% (*n* = 34) of the participants completed all modules. The self-estimated number of times engaging in mindfulness sessions during the course of treatment was 18.7 (*SD* = 14.52, range 0-62) and the self-estimated time in minutes engaging in mindfulness was 73.68 minutes (*SD* = 78.76, range 0-376).

### Treatment Effects

Means and standard deviations including ANCOVA *F*-values and effect sizes are presented in Table 3. A large between-group effect size was found at posttreatment, *d* = 0.79, 95% CI [0.36, 1.23], on the primary outcome PSS-14 measuring perceived levels of stress. The secondary outcome measure KEDS assessing exhaustion-related symptoms demonstrated a similar change. The ANCOVA was statistically significant with a moderate effect size of *d* = 0.65, 95% CI [0.24, 1.06]. For the BBQ measuring quality of life the ANCOVA was statistically significant, but with a smaller effect size of *d* = 0.40, 95% CI [0.03, 0.81]. Regarding the FFMQ-SWE measuring mindfulness there was an effect in favor of the treatment group with a moderate effect size of *d* = 0.66, 95% CI [0.26, 1.07]. The reported increase in the level of mindfulness was positively correlated with both the number of training sessions (*rho* = .38, *p* = .016) and the total duration of training (*rho* = .31, *p* = .049). There was also a significant negative correlation between reported changes in the level of mindfulness and experienced level of stress, respectively. Those achieving a higher level of mindfulness reported a larger decrease in their stress level (*r* = .36, *p* = .023).

**Table 3:** Means (M), Standard Deviations (SD), Effect Sizes (Cohen’s d), and ANCOVA Results for Stress, Exhaustion, Quality of Life, and Mindfulness Measures in Treatment (n = 48) and Control (n = 49) Groups

Measure / Group	Pre		Post		Cohen's *d*	ANCOVA *F*(1, 96)
	*M*	*SD*	*M*	*SD*		
PSS					0.79	28.4***
Treatment	33.09	6.66	23.46	8.98		
Control	33.96	6.24	29.82	7.04		
KEDS					0.65	25.3***
Treatment	26.06	8.94	18.33	9.17		
Control	27.45	8.14	23.95	7.93		
BBQ					0.40	6.4*
Treatment	44.90	18.22	54.37	19.89		
Control	42.82	16.78	46.80	17.27		
FFMQ-SWE					0.66	19.9***
Treatment	83.79	12.00	93.18	9.60		
Control	84.12	9.40	86.10	11.42		

*Note.* ANCOVA = analysis of covariance; PSS = Perceived Stress Scale; KEDS = Karolinska Exhaustion Disorder Scale; BBQ = Brunnsviken Brief Quality of Life; FFMQ-SWE = Five Facets of Mindfulness, Swedish version.

*p < .05. ***p < .001.

## Discussion

The present study evaluated the effects of a mindfulness-focused ICBT program on elevated stress- and exhaustion symptoms, and quality of life. The study also investigated the effect of the number of mindfulness sessions and the total amount of training with regard to changes in the level of mindfulness, as well as the association between changes in the level of mindfulness and symptoms of stress. The study results show that the treatment group reduced their perceived levels of stress, such as experiences of not having control and feelings of being overloaded, significantly more than the waitlist control group. This is in line with previous research on CBT-based stress management (Richardson & Rothstein, 2008), mindfulness interventions (Khoury et al., 2013), and results from other ICBT-studies, showing that digital interventions for stress related problems can be effective with moderate to large effect sizes (Svärdman et al., 2022). Results also showed that the intervention group had significant effects in comparison with the control group on symptoms of exhaustions, such as memory, ability to concentrate and fatigue. In Sweden, exhaustion disorder accounts for more instances of long-term sick leave than any other diagnose and there is a limited amount of treatment research and limited evidence (Lindsäter et al., 2022) The findings in this study adds to the literature showing that ICBT interventions have the potential to influence work related outcomes such as levels of exhaustion (Asplund et al., 2023) and can be a cost-effective treatment option (Lindsäter et al., 2019). We also investigated if the treatment program would increase quality of life compared to the waitlist control group, which it did. The significant small to moderate effect on quality of life is in line with the effects usually obtained in CBT-studies on this measure (Kolovos et al., 2016).

Despite the short period of time for the intervention, participants in the treatment group increased their level of mindfulness significantly compared to the waitlist during the program. This is interesting as mindfulness is known to take long time and much effort to practice before effects can be shown (Brand et al., 2012). We found that the amount of mindfulness training, both number of sessions and total length, was significantly associated with increased self-reported mindfulness. This finding is confirmed by meta-analytic evidence showing that training in mindfulness can affect the dimensions of mindfulness captured by FFMQ (Quaglia et al., 2016). It should be mentioned that there could be a methodological problem as the measure of amount of mindfulness practice was self-estimated and could be influenced by social desirability.

We also found that the level of mindfulness was significantly associated with reduced levels of perceived stress symptoms. This may be because individuals who improve their ability to have a non-reactive approach to inner experiences, and the ability to observe and describe the present situation in a non-judging manner, experience less stress symptoms. This is in line with previous literature (Chiesa & Serretti, 2009; Shapiro et al., 2005; Smith et al., 2008) showing an effect of mindfulness techniques on reduced level of perceived stress symptoms in a clinical as well as a non-clinical population. Nevertheless, due to the absence of information regarding the direction of this association, it is equally plausible that reduced stress symptoms heighten individuals' awareness of their internal context.

Compared to other internet-based treatment programs, the intervention used in this study was less text-driven, shorter in length (length and number of modules) and developed to be interactive and motivating to work with for participants. There is indicative evidence that more condensed internet-based interventions are equally as effective (Karlsson-Good et al., 2023) and it could also be that the user experience had an effect on motivation and the use of treatment content, which is supported by the fact that the overall rating of the user experience was considered excellent by participants (Bangor et al., 2008). This topic needs to be further investigated, as the use of technology for increased effect and compliance in digital interventions are still under-researched (Balcombe & Leo, 2022; Wildeboer et al., 2016).

The limitations and strengths in the present study should be mentioned. First, there were no follow-up measurement. Thus, long-term effects were not measured which should be valuable to investigate in future studies. However, the robust design of RCT and the high number of individuals who completed the program are strengths in the study. The study also has high generalizability to the clinical population as the inclusion criteria for individuals recruited in the study were broad and there were no exclusions of individuals due to comorbidity. A possible limitation is the inclusion of a subclinical population as there are no norms available for PSS-14 and we did not exclude participants based on a certain lower threshold on the scale, although the data from this sample shows that the participants experienced elevated levels of perceived stress and exhaustion comparable to what is seen in other studies (Asplund et al., 2018). Other limitations are the possibility of selection bias using an open recruitment strategy and that we have no information on the direction of the associations being studied and they should therefore be interpreted with caution.

The present randomized controlled study provides knowledge that a mindfulness-focused ICBT stress program can reduce perceived stress and symptoms of exhaustion, and also increase quality of life and the experience of mindfulness. This shows that short internet-based interventions combining CBT and mindfulness have the potential to lessen the burden of stress-related problems.

## Data Availability

Data, material and analysis methods from the trial can be made available for other researchers upon request.

## Preregistration

The trial was not preregistered.

## Reporting Guidelines

The study followed Consolidated Standards of Reporting Trials (CONSORT) guidelines.

## References

[r1] Andersson, G., Carlbring, P., Titov, N., & Lindefors, N. (2019). Internet interventions for adults with anxiety and mood disorders: A narrative umbrella review of recent meta-analyses. Canadian Journal of Psychiatry, 64(7), 465–470. 10.1177/0706743719839381 31096757 PMC6610559

[r2] Andersson, G., Rozental, A., Shafran, R., & Carlbring, P. (2018). Long-term effects of internet-supported cognitive behaviour therapy. Expert Review of Neurotherapeutics, 18(1), 21–28. 10.1080/14737175.2018.1400381 29094622

[r3] Andersson, G., Titov, N., Dear, B. F., Rozental, A., & Carlbring, P. (2019). Internet-delivered psychological treatments: From innovation to implementation. World Psychiatry, 18(1), 20–28. 10.1002/wps.20610 30600624 PMC6313242

[r4] Asplund, R. P., Asplund, S., von Buxhoeveden, H., Delby, H., Eriksson, K., Gerhardsson, M. S., Palm, J., Skyttberg, T., Torstensson, J., Ljótsson, B., Carlbring, P., & Andersson, G. (2023). Work-focused versus generic internet-based interventions for employees with stress-related disorders: Randomized controlled trial. Journal of Medical Internet Research, 25, e34446. 10.2196/34446 37097739 PMC10170369

[r5] Asplund, R. P., Dagöö, J., Fjellström, I., Niemi, L., Hansson, K., Zeraati, F., Ziuzina, M., Geraedts, A., Ljótsson, B., Carlbring, P., & Andersson, G. (2018). Internet-based stress management for distressed managers: Results from a randomised controlled trial. Occupational and Environmental Medicine, 75(2), 105–113. 10.1136/oemed-2017-104458 28855344 PMC5800342

[r6] Atroszko, P. A., Demetrovics, Z., & Griffiths, M. D. (2020). Work addiction, obsessive-compulsive personality disorder, burn-out, and global burden of disease: Implications from the ICD-11. International Journal of Environmental Research and Public Health, 17(2), 660. 10.3390/ijerph17020660 31968540 PMC7014139

[r7] Baer, R. A., Smith, G. T., Lykins, E., Button, D., Krietemeyer, J., Sauer, S., Walsh, E., Duggan, D., & Williams, J. M. G. (2008). Construct validity of the Five Facet Mindfulness Questionnaire in meditating and nonmeditating samples. Assessment, 15(3), 329–342. 10.1177/1073191107313003 18310597

[r8] Balcombe, L., & Leo, D. D. (2022). Human-computer interaction in digital mental health. Informatics, 9(1), 14. 10.3390/informatics9010014

[r9] Bangor, A., Kortum, P. T., & Miller, J. T. (2008). An empirical evaluation of the System Usability Scale. International Journal of Human-Computer Interaction, 24(6), 574–594. 10.1080/10447310802205776

[r10] Besèr, A., Sorjonen, K., Wahlberg, K., Peterson, U., Nygren, Å., & Åsberg, M. (2014). Construction and evaluation of a self rating scale for stress‐induced exhaustion disorder, the Karolinska Exhaustion Disorder Scale. Scandinavian Journal of Psychology, 55(1), 72–82. 10.1111/sjop.12088 24236500 PMC4235404

[r11] Bhattacharya, S., Goicoechea, C., Heshmati, S., Carpenter, J. K., & Hofmann, S. G. (2023). Efficacy of cognitive behavioral therapy for anxiety-related disorders: A meta-analysis of recent literature. Current Psychiatry Reports, 25(1), 19–30. 10.1007/s11920-022-01402-8 36534317 PMC9834105

[r12] Bhui, K. S., Dinos, S., Stansfeld, S. A., & White, P. D. (2012). A synthesis of the evidence for managing stress at work: A review of the reviews reporting on anxiety, depression, and absenteeism. Journal of Environmental and Public Health, 2012, 515874. 10.1155/2012/515874 22496705 PMC3306941

[r13] Bishop, S. R., Lau, M., Shapiro, S., Carlson, L., Anderson, N. D., Carmody, J., Segal, Z. V., Abbey, S., Speca, M., Velting, D., & Devins, G. (2004). Mindfulness: A proposed operational definition. Clinical Psychology: Science and Practice, 11(3), 230–241. 10.1093/clipsy.bph077

[r14] Brand, S., Holsboer-Trachsler, E., Naranjo, J. R., & Schmidt, S. (2012). Influence of mindfulness practice on cortisol and sleep in long-term and short-term meditators. Neuropsychobiology, 65(3), 109–118. 10.1159/000330362 22377965

[r15] Brooke, J. (1996). SUS: A “quick and dirty” usability scale. In P. Jordan, B. Thomas, & B. Weerdmeester (Eds.), *Usability evaluation in industry* (pp. 189–194). Taylor & Francis.

[r16] Butler, A. C., Chapman, J. E., Forman, E. M., & Beck, A. T. (2006). The empirical status of cognitive-behavioral therapy: A review of meta-analyses. Clinical Psychology Review, 26(1), 17–31. 10.1016/j.cpr.2005.07.003 16199119

[r17] Carlbring, P., Hägglund, M., Luthström, A., Dahlin, M., Kadowaki, Å., Vernmark, K., & Andersson, G. (2013). Internet-based behavioral activation and acceptance-based treatment for depression: A randomized controlled trial. Journal of Affective Disorders, 148(2–3), 331–337. 10.1016/j.jad.2012.12.020 23357657

[r18] Catarino, A., Harper, S., Malcolm, R., Stainthorpe, A., Warren, G., Margoum, M., Hooper, J., Blackwell, A. D., & Welchman, A. E. (2023). Economic evaluation of 27,540 patients with mood and anxiety disorders and the importance of waiting time and clinical effectiveness in mental healthcare. Nature Mental Health, 1(9), 667–678. 10.1038/s44220-023-00106-z

[r19] Chiesa, A., & Serretti, A. (2009). Mindfulness-based stress reduction for stress management in healthy people: A review and meta-analysis. Journal of Alternative and Complementary Medicine, 15(5), 593–600. 10.1089/acm.2008.0495 19432513

[r20] Christie, A. M., Atkins, P. W. B., & Donald, J. N. (2017). The meaning and doing of mindfulness: The role of values in the link between mindfulness and well-being. Mindfulness, 8(2), 368–378. 10.1007/s12671-016-0606-9

[r21] Cohen, S., Janicki-Deverts, D., & Miller, G. E. (2007). Psychological stress and disease. Journal of the American Medical Association, 298(14), 1685–1687. 10.1001/jama.298.14.1685 17925521

[r22] Cohen, S., Kamarck, T., & Mermelstein, R. (1983). A global measure of perceived stress. Journal of Health and Social Behavior, 24(4), 385–396. 10.2307/2136404 6668417

[r23] Deshpande, A. G., Johnson, J. R., Casta, A. M., Marien, M. S., & Reiff, M. (2023). The impact of a mindfulness-based stress reduction program on university students’ mental health: A mixed-methods evaluation. Journal of American College Health. Advance online publication. 10.1080/07448481.2023.2198028 37053589

[r24] Ebert, D. D., Lehr, D., Heber, E., Riper, H., Cuijpers, P., & Berking, M. (2016). Internet- and mobile-based stress management for employees with adherence-focused guidance: Efficacy and mechanism of change. Scandinavian Journal of Work, Environment & Health, 42(5), 382–394. 10.5271/sjweh.3573 27249161

[r25] Eklund, M., Bäckström, M., & Tuvesson, H. (2014). Psychometric properties and factor structure of the Swedish version of the Perceived Stress Scale. Nordic Journal of Psychiatry, 68(7), 494–499. 10.3109/08039488.2013.877072 24460116

[r26] Enders, C. K. (2017). Multiple imputation as a flexible tool for missing data handling in clinical research. Behaviour Research and Therapy, 98, 4–18. 10.1016/j.brat.2016.11.008 27890222

[r27] Ghazavi, Z., Rahimi, E., Yazdani, M., & Afshar, H. (2016). Effect of cognitive behavioral stress management program on psychosomatic patients’ quality of life. Iranian Journal of Nursing and Midwifery Research, 21(5), 510–515. 10.4103/1735-9066.193415 27904636 PMC5114797

[r28] Glise, K., Hadzibajramovic, E., Jonsdottir, I. H., & Ahlborg, G. (2010). Self-reported exhaustion: A possible indicator of reduced work ability and increased risk of sickness absence among human service workers. International Archives of Occupational and Environmental Health, 83(5), 511–520. 10.1007/s00420-009-0490-x 19943058

[r29] González-Ramírez, M. T., Rodríguez-Ayán, M. N., & Hernández, R. L. (2013). The Perceived Stress Scale (PSS): Normative data and factor structure for a large-scale sample in Mexico. The Spanish Journal of Psychology, 16, E47. 10.1017/sjp.2013.35 23866243

[r30] Grossi, G., Perski, A., Osika, W., & Savic, I. (2015). Stress‐related exhaustion disorder – Clinical manifestation of burnout? A review of assessment methods, sleep impairments, cognitive disturbances, and neuro‐biological and physiological changes in clinical burnout. Scandinavian Journal of Psychology, 56(6), 626–636. 10.1111/sjop.12251 26496458

[r31] Hassard, J., Teoh, K. R. H., Visockaite, G., Dewe, P., & Cox, T. (2018). The cost of work-related stress to society: A systematic review. Journal of Occupational Health Psychology, 23(1), 1–17. 10.1037/ocp0000069 28358567

[r32] Hayes, S. C. (2016). Acceptance and commitment therapy, relational frame theory, and the third wave of behavioral and cognitive therapies – Republished article. Behavior Therapy, 47(6), 869–885. 10.1016/j.beth.2016.11.006 27993338

[r33] Heber, E., Ebert, D. D., Lehr, D., Cuijpers, P., Berking, M., Nobis, S., & Riper, H. (2017). The benefit of web- and computer-based interventions for stress: A systematic review and meta-analysis. Journal of Medical Internet Research, 19(2), e32. 10.2196/jmir.5774 28213341 PMC5336602

[r34] Heber, E., Lehr, D., Ebert, D. D., Berking, M., & Riper, H. (2016). Web-based and mobile stress management intervention for employees: A randomized controlled trial. Journal of Medical Internet Research, 18(1), e21. 10.2196/jmir.5112 26818683 PMC4749847

[r35] Hedman‐Lagerlöf, E., Carlbring, P., Svärdman, F., Riper, H., Cuijpers, P., & Andersson, G. (2023). Therapist‐supported Internet‐based cognitive behaviour therapy yields similar effects as face‐to‐face therapy for psychiatric and somatic disorders: An updated systematic review and meta‐analysis. World Psychiatry, 22(2), 305–314. 10.1002/wps.21088 37159350 PMC10168168

[r36] Hepburn, S.-J., Carroll, A., & McCuaig, L. (2021). The relationship between mindful attention awareness, perceived stress and subjective wellbeing. International Journal of Environmental Research and Public Health, 18(23), 12290. 10.3390/ijerph182312290 34886026 PMC8656828

[r37] Höglund, P., Hakelind, C., & Nordin, S. (2020). Severity and prevalence of various types of mental ill-health in a general adult population: Age and sex differences. BMC Psychiatry, 20, 209. 10.1186/s12888-020-02557-5 32393209 PMC7212684

[r38] Hvidt, J. C. S., Christensen, L. F., Sibbersen, C., Helweg-Jørgensen, S., Hansen, J. P., & Lichtenstein, M. B. (2020). Translation and validation of the System Usability Scale in a Danish mental health setting using digital technologies in treatment interventions. International Journal of Human-Computer Interaction, 36(8), 709–716. 10.1080/10447318.2019.1680922

[r39] Janssen, M., Heerkens, Y., Kuijer, W., van der Heijden, B., & Engels, J. (2018). Effects of Mindfulness-Based Stress Reduction on employees’ mental health: A systematic review. PLoS One, 13(1), e0191332. 10.1371/journal.pone.0191332 29364935 PMC5783379

[r40] Javaid, Z. K., Mahmood, K., & Ali, A. A. (2023). Mediating role of mindfulness between quality of life and workplace stress among working women: Quality of life and workplace stress among working women. Journal of Workplace Behavior, 4(1), 68–80. https://charisma-jwb.com/index.php/jwb/article/view/170 10.25215/0604.079

[r41] Kabat‐Zinn, J. (2003). Mindfulness‐based interventions in context: Past, present, and future. Clinical Psychology: Science and Practice, 10(2), 144–156. 10.1093/clipsy.bpg016

[r42] Karlsson-Good, M., Kaldo, V., Lundberg, L., Kraepelien, M., Anthony, S. A., & Holländare, F. (2023). Increasing the accessibility to internet-based cognitive behavioural therapy for depression: A single-blind randomized controlled trial of condensed versus full-text versions. Internet Interventions, 34, 100678. 10.1016/j.invent.2023.100678 37840646 PMC10570001

[r43] Khoury, B., Lecomte, T., Fortin, G., Masse, M., Therien, P., Bouchard, V., Chapleau, M.-A., Paquin, K., & Hofmann, S. G. (2013). Mindfulness-based therapy: A comprehensive meta-analysis. Clinical Psychology Review, 33(6), 763–771. 10.1016/j.cpr.2013.05.005 23796855

[r44] Kivimäki, M., & Steptoe, A. (2018). Effects of stress on the development and progression of cardiovascular disease. Nature Reviews Cardiology, 15(4), 215–229. 10.1038/nrcardio.2017.189 29213140

[r45] Kolovos, S., Kleiboer, A., & Cuijpers, P. (2016). Effect of psychotherapy for depression on quality of life: Meta-analysis. The British Journal of Psychiatry, 209(6), 460–468. 10.1192/bjp.bp.115.175059 27539296

[r46] Lilja, J. L., Frodi-Lundgren, A., Hanse, J. J., Josefsson, T., Lundh, L.-G., Sköld, C., Hansen, E., & Broberg, A. G. (2011). Five Facets Mindfulness Questionnaire—Reliability and Factor structure: A Swedish version. Cognitive Behaviour Therapy, 40(4), 291–303. 10.1080/16506073.2011.580367 21770845

[r47] Lindner, P., Frykheden, O., Forsström, D., Andersson, E., Ljótsson, B., Hedman, E., Andersson, G., & Carlbring, P. (2016). The Brunnsviken Brief Quality of Life Scale (BBQ): Development and psychometric evaluation. Cognitive Behaviour Therapy, 45(3), 182–195. 10.1080/16506073.2016.1143526 26886248 PMC4867878

[r48] Lindsäter, E., Axelsson, E., Salomonsson, S., Santoft, F., Ljótsson, B., Åkerstedt, T., Lekander, M., & Hedman-Lagerlöf, E. (2019). Cost-effectiveness of therapist-guided internet-based cognitive behavioral therapy for stress-related disorders: Secondary analysis of a randomized controlled trial. Journal of Medical Internet Research, 21(9), e14675. 10.2196/14675 31586370 PMC6788336

[r49] Lindsäter, E., Svärdman, F., Wallert, J., Ivanova, E., Söderholm, A., Fondberg, R., Nilsonne, G., Cervenka, S., Lekander, M., & Rück, C. (2022). Exhaustion disorder: Scoping review of research on a recently introduced stress-related diagnosis. BJPsych Open, 8(5), e159. 10.1192/bjo.2022.559 36458830 PMC9438479

[r50] Ly, K. H., Asplund, K., & Andersson, G. (2014). Stress management for middle managers via an acceptance and commitment-based smartphone application: A randomized controlled trial. Internet Interventions, 1(3), 95–101. 10.1016/j.invent.2014.06.003

[r51] Melchior, M., Caspi, A., Milne, B. J., Danese, A., Poulton, R., & Moffitt, T. E. (2007). Work stress precipitates depression and anxiety in young, working women and men. Psychological Medicine, 37(8), 1119–1129. 10.1017/S0033291707000414 17407618 PMC2062493

[r52] Michel, A., Bosch, C., & Rexroth, M. (2014). Mindfulness as a cognitive–emotional segmentation strategy: An intervention promoting work–life balance. Journal of Occupational and Organizational Psychology, 87(4), 733–754. 10.1111/joop.12072

[r53] Myhr, G., & Payne, K. (2006). Cost-effectiveness of cognitive-behavioural therapy for mental disorders: Implications for public health care funding policy in Canada. Canadian Journal of Psychiatry, 51(10), 662–670. 10.1177/070674370605101006 17052034

[r54] Nakao, M., Shirotsuki, K., & Sugaya, N. (2021). Cognitive–behavioral therapy for management of mental health and stress-related disorders: Recent advances in techniques and technologies. BioPsychoSocial Medicine, 15(1), 16. 10.1186/s13030-021-00219-w 34602086 PMC8489050

[r55] Parsaei, R., Roohafza, H., Feizi, A., Sadeghi, M., & Sarrafzadegan, N. (2020). How different stressors affect quality of life: An application of multilevel latent class analysis on a large sample of industrial employees. Risk Management and Healthcare Policy, 13, 1261–1270. 10.2147/RMHP.S256800 32903876 PMC7445524

[r56] Quaglia, J. T., Braun, S. E., Freeman, S. P., McDaniel, M. A., & Brown, K. W. (2016). Meta-analytic evidence for effects of mindfulness training on dimensions of self-reported dispositional mindfulness. Psychological Assessment, 28(7), 803–818. 10.1037/pas0000268 27078183

[r57] Richardson, K. M., & Rothstein, H. R. (2008). Effects of occupational stress management intervention programs: A meta-analysis. Journal of Occupational Health Psychology, 13(1), 69–93. 10.1037/1076-8998.13.1.69 18211170

[r58] Schulz, K. F., Altman, D. G., Moher, D., & Group, C. (2010). CONSORT 2010 Statement: Updated guidelines for reporting parallel group randomised trials. BMC Medicine, 8(1), 18. 10.1186/1741-7015-8-18 20334633 PMC2860339

[r59] Shapiro, S. L., Astin, J. A., Bishop, S. R., & Cordova, M. (2005). Mindfulness-based stress reduction for health care professionals: Results from a randomized trial. International Journal of Stress Management, 12(2), 164–176. 10.1037/1072-5245.12.2.164

[r60] Smith, B. W., Shelley, B. M., Dalen, J., Wiggins, K., Tooley, E., & Bernard, J. (2008). A pilot study comparing the effects of mindfulness-based and cognitive-behavioral stress reduction. Journal of Alternative and Complementary Medicine, 14(3), 251–258. 10.1089/acm.2007.0641 18370583

[r61] Svanborg, P., & Åsberg, M. (1994). A new self-rating scale for depression and anxiety states based on the Comprehensive Psychopathological Rating Scale. Acta Psychiatrica Scandinavica, 89(1), 21–28. 10.1111/j.1600-0447.1994.tb01480.x 8140903

[r62] Svanborg, P., & Åsberg, M. (2001). A comparison between the Beck Depression Inventory (BDI) and the self-rating version of the Montgomery Åsberg Depression Rating Scale (MADRS). Journal of Affective Disorders, 64(2–3), 203–216. 10.1016/S0165-0327(00)00242-1 11313087

[r63] Svärdman, F., Sjöwall, D., & Lindsäter, E. (2022). Internet-delivered cognitive behavioral interventions to reduce elevated stress: A systematic review and meta-analysis. Internet Interventions, 29, 100553. 10.1016/j.invent.2022.100553 35781929 PMC9240371

[r64] Sverre, K. T., Nissen, E. R., Farver-Vestergaard, I., Johannsen, M., & Zachariae, R. (2023). Comparing the efficacy of mindfulness-based therapy and cognitive-behavioral therapy for depression in head-to-head randomized controlled trials: A systematic review and meta-analysis of equivalence. Clinical Psychology Review, 100, 102234. 10.1016/j.cpr.2022.102234 36527794

[r65] van Dam, A. (2021). A clinical perspective on burnout: Diagnosis, classification, and treatment of clinical burnout. European Journal of Work and Organizational Psychology, 30(5), 732–741. 10.1080/1359432X.2021.1948400

[r66] Vickers, A. J., & Altman, D. G. (2001). Analysing controlled trials with baseline and follow up measurements. BMJ, 323(7321), 1123. 10.1136/bmj.323.7321.1123 11701584 PMC1121605

[r67] Weineland, S., Ribbegårdh, R., Kivi, M., Bygdell, A., Larsson, A., Vernmark, K., & Lilja, J. L. (2020). Transitioning from face-to-face treatment to iCBT for youths in primary care – Therapists’ attitudes and experiences. Internet Interventions, 22, 100356. 10.1016/j.invent.2020.100356 33318951 PMC7724368

[r68] Wenzel, A., Dobson, K. S., & Hays, P. A. (2016). Introduction. In *Cognitive behavioral therapy techniques and strategies* (pp. 3–14). 10.1037/14936-001

[r69] Wildeboer, G., Kelders, S. M., & van Gemert-Pijnen, J. E. W. C. (2016). The relationship between persuasive technology principles, adherence and effect of web-based interventions for mental health: A meta-analysis. International Journal of Medical Informatics, 96, 71–85. 10.1016/j.ijmedinf.2016.04.005 27117057

[r70] Zetterqvist, K., Maanmies, J., Ström, L., & Andersson, G. (2003). Randomized controlled trial of internet-based stress management. Cognitive Behaviour Therapy, 32(3), 151–160. 10.1080/16506070302316 16291546

